# Immune-Inflammatory Responses in Atherosclerosis: The Role of Myeloid Cells

**DOI:** 10.3390/jcm8111798

**Published:** 2019-10-27

**Authors:** Dimitry A. Chistiakov, Dmitry A. Kashirskikh, Victoriya A. Khotina, Andrey V. Grechko, Alexander N. Orekhov

**Affiliations:** 1 Laboratory of Angiopathology, Institute of General Pathology and Pathophysiology, 125315 Moscow, Russia; dimitry.chistiakov@lycos.com (D.A.C.); dim.kashirsckih@gmail.com (D.A.K.); nafany905@gmail.com (V.A.K.); 2 Federal Research and Clinical Center of Intensive Care Medicine and Rehabilitology, 109240 Moscow, Russia; noo@fnkcrr.ru; 3 Institute of Human Morphology, Tsyrupa st. 3, 117418 Moscow, Russia

**Keywords:** Atherosclerosis, inflammation, myeloid cell, macrophage, dendritic cell

## Abstract

Inflammation plays a key role in the initiation and progression of atherosclerosis and can be caused by multiple agents, including increased concentration of circulating low-density lipoprotein (LDL) cholesterol. Areas of the arterial wall affected by atherosclerosis are enriched with lymphocytes and dendritic cells (DCs). Atherosclerotic plaques contain a variety of proinflammatory immune cells, such as macrophages, DCs, T cells, natural killer cells, neutrophils and others. Intracellular lipid accumulation in atherosclerotic plaque leads to formation of so-called foam cells, the cytoplasm of which is filled with lipid droplets. According to current understanding, these cells can also derive from the immune cells that engulf lipids by means of phagocytosis. Macrophages play a crucial role in the initial stages of atherogenesis by engulfing oxidized LDL (oxLDL) in the intima that leads to their transformation to foam cells. Dying macrophages inside the plaque form a necrotic core that further aggravates the lesion. Proinflammatory DCs prime differentiation of naïve T cells to proinflammatory Th1 and Th17 subsets. In this review, we discuss the roles of cell types of myeloid origin in atherosclerosis-associated inflammation.

## 1. Introduction

Atherosclerosis is a common pathology that affects large and medium arteries leading to a variety of cardiovascular events that can also be fatal, therefore representing a serious public health problem. Atherosclerosis is characterized by progressive lipid deposition in the arterial wall, plaque induction and possible further progression to the state of so-called unstable plaque. Advanced plaques usually acquire a fibrous cap that separates the plaque from the surrounding milieu and plays a stabilizing role. Destabilization of plaques leads to their rupture with subsequent thrombus formation that can lead to dangerous events such as acute coronary syndrome (ACS) and stroke [1]. Advanced complicated plaques are also characterized by the formation of the necrotic core, pathological vascular remodeling, plaque neovascularization and calcification.

Elevated low-density lipoprotein (LDL) is known to be associated with atherosclerosis and is identified as the major source of lipids accumulating in atherosclerotic plaques. Circulating modified LDL has increased atherogenicity in comparison to native LDL. Chemical modification of LDL likely plays a prominent role in atherosclerosis development [2]. Besides elevated LDL, chronic inflammation is now considered as another crucial pro-atherogenic mechanism that can even precede lipid entry into the arterial intima [3]. 

It has been proposed that disturbances of hemodynamic forces that are especially likely to occur at atheroprone sites of blood vessels, such as bends or bifurcations, can cause local activation of endothelial cells (ECs). Consequently, ECs acquire a pro-inflammatory phenotype and start expressing different pro-inflammatory molecules, such as monocyte chemotactic protein 1 (MCP1) (also known as chemokine (C-C motif) ligand 2; CCL2), intercellular adhesion molecule 1 (ICAM-1) and vascular cell adhesion molecule 1 (VCAM-1), all of which attract monocytes to the activated endothelium [4]. The imbalance of monocyte/macrophage polarization towards the preferential pro-inflammatory phenotype and a lack of normal inflammation resolution are also present in atherosclerosis [5]. Phagocytic macrophages actively participate in lipid accumulation by engulfing lipid droplets and give rise to foam cells. Dendritic cells (DCs) contribute to the local inflammatory response through antigen presentation and cytokine signaling processes [6]. If the inflammatory process reaches a resolution, repair processes begin, and the plaque can become stable. However, failure to resolve the inflammatory response can result in the formation of the most dangerous unstable plaque that can cause thrombosis.

Therefore, a variety of immune cells are involved in atherosclerosis initiation and progression. In this review, we consider main subsets of pro- and anti-inflammatory immune cells of myeloid origin that contribute to atherogenesis.

## 2. Myeloid Immune Cells Involved in Atherosclerosis

Presence of leukocytes in blood vessels affected by atherosclerosis was first described in late 1970s [7]. Along with macrophages, other common leukocyte types were reported to be present in affected arteries [8]. Presence of pro-inflammatory cells in atherosclerotic plaques depends on multiple factors. One of the most important of them is the degree of immune recruitment and retention, cell proliferation rate and apoptosis/necrosis. Leukocytes can be recruited quickly but have a long-term residence in the plaque [6].

In C57BL/6 mice, macrophages and DCs were detected in the arterial wall at the pre-atherosclerotic stage. Macrophages were present across the adventitia whereas DCs were detected in the intima of atherosclerosis-prone regions [9]. In the plaques of apolipoprotein E (ApoE)-deficient mice, macrophage-derived foam cells were shown to arise from circulating precursors rather from resident macrophages [10]. Similarly, in humans, increased numbers of DCs were observed in atheroprone regions of blood vessels from apparently healthy subjects [11]. This phenomenon could be explained by DC attraction to the regions of inflamed endothelium that releases CCL2 (an attractant for DCs) [12] and expresses major histocompatibility complex (MHC) molecules on its surface, thereby participating in antigen presentation [13]. 

Monocyte recruitment is a multistep process that includes tethering, rolling, adhesion and transmigration to the subendothelial layer [14]. The rolling stage is directed by P- and E-selectins that are present on the surface of ECs. Monocytes have a surface selectin P ligand (SELPG, also known as CD162) that interacts with both endothelial selectins [15]. Another regulator of slow rolling and tight adhesion of monocytes is VCAM-1, while ICAM-1 seems to play a secondary role in monocyte adhesion. Integrin α4β1, which is expressed on the surface of monocytes, can interact with VCAM-1 and slow the rolling. A number of chemokine ligands such as CXCL1, CXCL2, CXCL4 and CCL5 expressed on the endothelial surface contribute to the monocyte rolling and increase α4β1-mediated adhesion through inside-out signaling and receptor clustering [7,8].

### 2.1. Monocytes

Monocytes belong to the myeloid lineage and are direct precursors of macrophages and monocyte-derived DCs. In response to pro-inflammatory stimuli, they rapidly migrate to the inflamed sites, where they differentiate predominantly to macrophages. In mice, inflammatory monocytes that express Ly6C in normal conditions are involved in the inflammatory response to microbial infections [16]. Ly6C is a monocyte/macrophage and EC differentiation antigen regulated by interferon (IFN)-γ, which is important for lymphocyte development and maturation [17]. C-C chemokine receptor type 2 (CCR2) binds CCL2 and is necessary for the induction of the predominant pro-inflammatory polarization of monocytes [18]. CX3C chemokine receptor 1 (CX3CR1) interacts with chemokine CX3CL1 (also known as fractalkine), which regulates migration and adhesion of monocytes [19]. Murine pro-inflammatory monocytes have phenotype Ly6C^high^CCR2^+^CX3CR1^low^ Notably, both CCL2 and CX3CR1 are linked to progression of atherosclerotic plaques [20].

Relatively recently, an unusual patrolling function of monocytes has been described [21]. Like macrophages and DCs, monocytes patrol the vascular endothelium under normal and inflammatory conditions in order to remove damaged cells and debris from the vasculature. They play a protective role and contribute to curing vascular injury and resolving inflammation. In mice and humans, patrolling monocytes have phenotypes CX3CR1^high^Ly6C^−^ (mice) and CX3CR1^high^CD14^dim^CD16^+^ (humans) that are different from the phenotypes of classical monocyte subpopulations (CCR2^high^Ly6C^+^ in mouse and CCR2^high^CD14^+^CD16^−^ in humans) [22]. The role of patrolling monocytes in atherosclerosis remains to be evaluated. 

Ly6^high^ monocytes predominantly move to atherosclerosis-susceptible arteries where they preferentially differentiate to pro-inflammatory macrophages through CX3CR1, CCR2 and CCR5 signaling [20]. The pro-atherosclerotic role of this trio was shown in apoE-deficient mice when joint suppression of these receptors abolished monocytosis and almost completely (up to 90%) diminished atherosclerosis [23]. By contrast, Ly6^low^ monocytes are probably needed only in CCR5 to migrate to the aorta [20]. 

### 2.2. Macrophages

Macrophages arise from monocytes and are actively participating in phagocytosis of bacteria, cellular debris, foreign particles, apoptotic/necrotic cells, cancer cells and other harmful agents. Macrophages represent the first line of the immune defense. Macrophages were the first immune cells found in atherosclerotic plaques: In 1979, Gerrity et al. first reported the presence of large numbers of macrophages in porcine lesions [6]. 

Macrophages play an important role in atherosclerosis-associated inflammation. They express scavenger receptors (SRs) and Toll-like receptors (TLR) that connect innate and adaptive immunity. In the plaque, macrophages take up modified atherogenic LDL and transform them into foam cells that are responsible for intracellular lipid accumulation in the arterial wall. Moreover, they release inflammatory cytokines and contribute to vascular remodeling [24]. The uptake of lipids by macrophages is enhanced after stimulation of TLR2, TLR4 and TLR9 by corresponding ligands, making TLRs important for atheroma development [25,26]. Genetic deletion of TLR2 and TLR4 resulted in a significant reduction (by 45% and 75%, respectively) of intimal lipid deposits in apoE-deficient mice, suggesting a pro-atherogenic role of these TLRs [27]. In macrophages, TLR3, TLR4 and TLR9 stimulate expression of SRs that play a crucial role in LDL intake [28]. In addition, activation of these TLRs leads to the suppression of cholesterol efflux from macrophages, which in turn favors lipid accumulation and foam cells formation [29]. 

The results of early studies indicated a strong pro-atherosclerotic role of SRs. For instance, knock-out of CD36 in apoE-deficient mice had an atheroprotective effect [30]. However, in subsequent studies, deletion of both SR-A1 and CD36 in apoE-deficient mice did not reduce atherosclerosis [31,32], suggesting the existence of alternative mechanisms of lipid uptake. One such alternative pathway could be mediated by lectin-like oxidized low-density lipoprotein receptor-1 (LOX-1). While CD36 binds modified oxLDL, LOX-1 interacts with other, moderately modified LDL forms, and can therefore be important at early stages of atherosclerosis development [33]. LOX-1 is normally not expressed in monocytes, however its expression can be induced in differentiated macrophages [34]. In atherosclerosis, LOX-1 was found to be significantly up-regulated in macrophages [35]. Knocking-down LOX-1 in non-stimulated macrophages had no great impact on oxLDL uptake, while in pro-inflammatory macrophages the effect was more pronounced, since the contribution of LOX-1 to total oxLDL uptake increased from 5% to 10%, to up to 40% [36]. In LDL receptor (LDLR)-deficient mice, LOX-1 knock-out had an anti-atherosclerotic effect, while its overexpression enhanced disease progression [37,38]. It can be therefore concluded that LOX-1 has a strong pro-atherogenic role.

**Figure 1:**
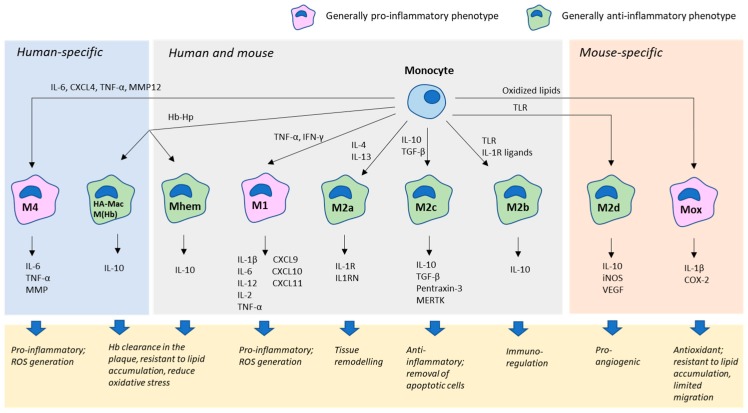
Macrophage diversity in the atherosclerotic plaque.

Macrophages are characterized by great plasticity, being able to adapt several different phenotypes [39]. Macrophage phenotype is a dynamic rather than terminally differentiated state, and can be changed in response to various signals [40]. Two major subtypes of macrophages that have been distinguished are the classical, pro-inflammatory M1 and alternative M2, which is responsible for tissue homeostasis and repair. Both subsets can be found in atherosclerotic plaques but play opposite roles (Figure 1). Formation of the classical M1 phenotype is induced by inflammatory cytokine signaling (such as tumor necrosis factor (TNF)-α and interferon (IFN)-γ), as well as by invading pathogens [41,42]. M1 macrophages have pro-inflammatory properties, releasing inflammatory cytokines IL-1β, IL-6, IL-12, IL-23 and TNF-α and chemokines CXCL9, CXCL10 and CXCL11 [43]. M1 cells produce high levels of nitric oxide (NO) and reactive oxygen species (ROS) and participate in Th1-mediated immune response. By contrast, M2 macrophages play an anti-inflammatory role and are involved in Th2-dependent immune cascades. This macrophage subclass is induced by Th2-type cytokines and releases anti-inflammatory cytokine IL-10 [43].

The M2 subset of macrophages can be further divided into several subsets that play different roles in the tissue and are induced by different stimuli. The M2a subset differentiates in response to IL-4 and IL-13 signaling and highly expresses CD206, IL-1 receptor (IL-1R) 2 and IL-1R antagonist (IL1RN). These macrophages are involved in tissue remodeling. The M2b subset is induced by immune complexes, TLR agonists or IL-1R ligands. These macrophages express IL-10 and are involved in immunoregulation [44]. The M2c subset develops in response to IL-10 and transforming growth factor (TGF)-β and glucocorticoids, and expresses mannose receptor (MR) in humans and arginase-1 in mouse. These macrophages express pentraxin-3, IL-10 and TGF-β, as well as Mer receptor kinase (MERTK), have anti-inflammatory properties and are responsible for removing apoptotic cells and debris [45]. M2a, M2b and M2c macrophages can be found in both human and murine atherosclerotic lesions, while the M2d subset is specific for mice. This macrophage subpopulation differentiates in response to TLR agonists signaling through the adenosine A2A receptor (ADORA2A) [46]. Stimulation of this receptor inhibits the expression of pro-inflammatory cytokines and stimulates production of IL-10, inducible NO-synthase and vascular endothelial growth factor (VEGF), thereby inducing pro-angiogenic properties in M2d macrophages [47].

M4 macrophages are specific for humans. This subtype differentiates in response to platelet-derived chemokine CXCL4 and releases IL-6, TNF-α, and matrix metalloproteinase MMP12, therefore being pro-inflammatory [48,49]. M4 macrophages were considered to possess some pro-atherosclerotic properties. However, they appear to be resistant to excessive lipid uptake and therefore transform to foam cells at a minimal rate [50].

In murine atherosclerotic plaques, oxidized phospholipids can induce polarization of naïve M0 macrophages to a pro-atherogenic Mox subset. Mox macrophages can also be induced by oxidized phospholipids from M1 and M2 macrophages by the transcription factor nuclear factor (erythroid-derived 2)-like 2 (NFE2L2), which initiates expression of several antioxidant and detoxifying genes [51]. Mox macrophages are characterized by limited migratory and phagocytic activity.

Hemorrhagic atherosclerotic plaques contain special subtypes of macrophages HA-mac and M(Hb) in humans and Mhem in both humans and mice. HA-mac and M(Hb) phenotypes can be induced by Hb–Hp complexes and are involved in intraplaque Hb clearance [52]. These macrophages, especially the HA-mac phenotype, express CD163, which is needed for sensing Hb–Hp complexes. HA-mac macrophages are also characterized by low expression of Human Leukocyte Antigen (HLA)-DR on their surface [53]. M(Hb) macrophages highly express MR and liver X receptor (LXR)-α, a nuclear transcription factor that stimulates expression of ATP-binding cassette transporters ABCA1 and ABCG1. Both transporters mediate cholesterol efflux. Therefore, M(Hb) macrophages are highly resistant against foam cell formation [54]. Mhem macrophages are characterized by high expression of CD163 and heme-dependent activating transcription factor (ATF)-1 [55]. ATF1 drives the expression of HMOX-1 and LXR-β and, through them, other genes involved in cholesterol efflux. Correspondingly, Mhem macrophages show increased resistance to foam cell formation. Clearance of Hb reduces oxidative stress and is associated with IL-10 production by Mhem, HA-mac and M(Hb) macrophages [56]. It can be concluded that all the three described subsets of macrophages play antioxidant, anti-inflammatory and atheroprotective roles.

Macrophage differentiation is accompanied by profound metabolic changes within the cell. For instance, pro-inflammatory M1 macrophages utilize L-arginine to produce NO, which is catalyzed by inducible nitric oxide synthase (iNOS). Alternatively, activated M2 macrophages use a different metabolic route for L-arginine, processing it to L-proline by arginase. Moreover, M1 macrophages are more dependent on glycolysis, while M2 macrophages metabolize glucose by means of oxidative phosphorylation [57]. That can explain the elevated production of ROS by pro-inflammatory macrophages, in which the process of ATP production is uncoupled from mitochondrial metabolism [58]. Finally, cholesterol metabolism also undergoes alterations during macrophage polarization, which has a special importance for atherosclerosis development [59]. Accumulation of cholesterol in myeloid cells has been identified as an important pro-inflammatory factor that acts through Toll-like receptor signaling and inflammasome activation. Atherogenic modified LDL have pro-inflammatory properties and can influence monocyte/macrophage phenotype by altering the metabolic pathways of these cells. It was shown that stimulation of myeloid cells with modified LDL resulted in immunological training, a reprogramming of metabolic pathways, including the rates of glycolysis versus oxidative phosphorylation, and cholesterol metabolism [60]. This effect has been shown both in vitro and in vivo in animal models. Together, these observations highlight the importance of the balance between pro- and anti-inflammatory polarization of macrophages in atherosclerosis development. Modulating of metabolic pathways of these cells, for instance, with statins that help normalize cholesterol metabolism and antioxidants that counterpart ROS formation has already been used for atherosclerosis treatment for a long time. However, improvement of the specificity of these therapies is needed to make them more efficient. One promising direction of such improvement is the development of therapies that can selectively counterpart the oxidative stress at the mitochondria level [61]. 

### 2.3. Dendritic Cells 

The first detection of DCs in atherosclerotic plaques of a human aorta was reported by Bobryshev and Lord in 1995 [59]. It was shown that DCs formed a network in the arterial intima. DCs are specialized antigen-presenting cells (APCs) that present a variety of antigens to T cells inducing and maintaining the immune response and suppressing activation of T cells. The immunostimulatory or immunoinhibitory functions of DCs depends on the cytokine production profile and the repertoire of co-stimulatory molecules expressed on the surface. By TLR-dependent stimulation, immature DCs can be transformed to APCs capable to activate naïve T cells to effector T cells. Immune tolerance is induced by antigen presentation when stimulatory signal such as TLR activation is absent. Therefore, DCs serve as a link between the adaptive and innate immunity [60]. 

Two main DC subsets are present in human circulation: conventional (c)DCs and plasmacytoid (p)DCs. cDCs are of myeloid origin and express blood DC antigen (BDCA)-1 (or CD1c), CD11c, TLR2, TLR3, TLR4 and TLR5 on the surface [61]. After stimulation with bacterial constituents, such as bacterial DNA, LPS, peptidoglycans or flagellin, cDCs mainly release IL-12 [62].

pDCs express CD123 and BDCA-2 (or CD303). Their primary function is the recognition of viral products and the induction of an innate antiviral response. pDC can sense viral DNA and RNA via TLR-9 and TLR-7 [63]. pDC are robust producers of type I IFNs (IFN-I) in response to viral infection [64]. In humans, a minor population of DCs expressing CD11c and BDCA-3 (or CD141) but lacking CD123, BDCA-1 and BDCA-2 has also been described [65]. This population is a unique subset of cDCs that is characterized by secretion of IL-12 and IFN-β and a higher expression of TLR3. Compared with BDCA-1^+^ cDCs, this subset shows a more pronounced capacity to induce Th1 cells. CD11c^+^BDCA-3^+^ cDCs also recognize necrotic cell antigens and induce cytotoxic T-cell responses against dead cells [66]. 

Monocytes can differentiate to DCs under inflammatory conditions and upon stimulation with granulocyte-macrophage colony-stimulating factor (GM-CSF) and TLR4 ligands, such as LPS [23]. Monocyte-derived DCs are able to present and cross-present antigens [67]. Compared to healthy individuals, patients with late atherosclerotic complications such as acute coronary syndrome (ACS) and unstable angina had monocyte-derived DCs more sensitive to stimulation by GM-CSF and IL-4 [68,69]. In line with this observation, monocytes from patients with coronary artery disease (CAD) exhibited a more profound capacity to differentiate into activated DCs compared to monocytes from normal subjects [70].

Within the plaque, the pro-inflammatory microenvironment supports preferential differentiation of DC precursors and monocytes towards pro-inflammatory DCs that can prime differentiation of naïve T cells to pro-inflammatory Th1 and Th17 subsets [71]. In apoE-deficient mice, treatment with self-antigenic complexes, loaded DNA from CAD patients and antimicrobial peptide resulted in advanced atherosclerosis mainly due to the stimulation of pDCs [72]. Microscopic studies of human carotid arteries revealed that pDCs were located primarily in the shoulder regions of the atherosclerotic plaque. These pDCs were responsive to pathogen-derived molecules and CpG-containing oligodeoxynucleotides by increased secretion of IFN-I that mediated differentiation of naïve T cells to cytotoxic CD4^+^ T cells. Activated T cells were shown to effectively kill aortic smooth muscle cells (SMCs) [73]. IFN-α produced by DCs is a strongly pro-inflammatory cytokine that activates cryopyrin inflammasome and inflammasome-dependent caspase-1 that leads to pyroptotic cell death. In this process, IL-1β and IL-18 are released, further aggravating vascular inflammation [74]. Pro-inflammatory activated pDCs also secrete chemokines CXCL9, CXCL10, CCL3, CCL4 and CCL5 that direct migration of inflammatory cells to the inflamed sites [75].

The maturation stage seems play a key role in determining whether DCs will possess inflammatory/immunoactivating or immunoregulatory/immunosuppressive properties [76]. Advanced lesions contain mature DCs in contact with T cells [77,78]. In the arterial wall, DCs stimulate local T cells through antigen presentation stimulating production of pro-inflammatory cytokines and maintaining the chronic inflammatory process. Mature CD11c^+^CD80^+^CD86^+^ cDCs are involved in supporting atherosclerotic inflammation by producing pro-inflammatory cytokine IL-12. oxLDL was shown to induce differentiation of monocytes to IL-12-producing DCs [79]. In atherosclerotic lesions of LDLR-deficient mice, DCs were found to take up oxLDL and initiate the generation of foam cells [80]. Human and murine atherosclerotic lesions contain a population of CCL17^+^CD11^+^ cDCs that have immunosuppressive properties and can efficiently inhibit regulatory T cells (Tregs), thereby contributing to atherogenesis [81]. CCL17 is expressed by mature cDCs of myeloid origin and stimulates migration and recruitment of CD4^+^ T cells and Tregs to inflamed sites. In apoE-deficient mice, deletion of CCL17 leads to delayed atherosclerosis progression and reduced numbers of macrophages and T cells in plaques suggesting a pro-atherogenic role of this cytokine [81].

Along with pro-inflammatory DCs, atherosclerotic plaques can contain tolerogenic and anti-inflammatory DC subsets that have atheroprotective properties. In humans, a population of tolerogenic CD86^−^CD80^−^CD40^−^ cDCs capable to induce Tregs has been described. This population consisted of immature cells that lacked MHC expression [82]. In mice, monocyte-derived CD11^high^MHC^high^CD11b^−^CD103^+^ DCs were shown to possess immunoregulatory properties through induction of Tregs [83]. Induction of Tregs by tolerogenic DCs occurs through direct cell–cell contacts or IL-10 and TGF-β signaling [84]. The key role of TGF-β in maintaining DCs tolerogenicity was demonstrated by knocking out a TGF-β receptor, TGFBR2, in CD11^+^ DCs, which led to the formation of pro-inflammatory and pro-atherogenic CD11^+^CD8^+^ cells and the accelerated progression of atherosclerosis in apoE-deficient mice [85].

Tolerogenic pDCs can perform immune regulation through induction of programmed death-ligand 1 (PD-L1), an immunosuppressor that requires binding to the PD-1 receptor or B7.1 receptor to prevent T-cell receptor (TCR)-dependent activation of T cells [86]. Another pathway is activation of indoleamine-pyrrole 2,3-dioxygenase (IDO), an enzyme involved in L-tryptophan catabolism. However, phosphorylation of IDO leads to the induction of immune regulatory function that is necessary for the maintenance of the regulatory properties in pDCs [87]. In addition, IDO mediates formation of Tregs from naïve T cells [88]. IDO is also involved in the regulation of activating effects of CCL17 and CCL22, e.g., chemokines that are crucial for efficient interaction between DCs and Tregs. CCL17 and CCL22 are ligands for their receptors CCR4 and CCR8, respectively, which are expressed on the surface of Tregs [81]. The pro-atherogenic activity of IDO was evaluated in animal studies involving LDLR-deficient mice. Specific depletion of IDO-producing pDC resulted in increased CD4^+^ T-cell proliferation and elevated levels of IFN-γ in the circulation [89]. Tolerogenic pDC may utilize IDO-dependent mechanism to prevent proliferation and activity of proinflammatory T cells. 

Anti-inflammatory tolerogenic pDCs can be induced by immunogenic peptides such as *Mycobacterium bovis* BCG antigens or ApoE-derived peptide Ep1.B. In ApoE-deficient mice, these pDCs induced IL-10-producing Tregs, thereby decreasing vascular inflammation and eventual plaque size [90,91,92,93].

## 3. Conclusions

Atherogenesis is a complex lifetime process that involves both innate and acquired immune response and chronic inflammation. Atherosclerotic lesion development is tightly linked to alterations in the balance of pro- and anti-inflammatory myeloid cells at the level of the arterial wall. Inside the plaque, several distinct types of myeloid cells can be observed: Pro-inflammatory macrophages promote further inflammation and contribute to the development of instable plaques, while alternatively activated anti-inflammatory cells participate in inflammation resolution and tissue repair. Specialized macrophage subtypes are responsible for heme cleaning at the sites of intraplaque hemorrhage. Monocyte to macrophage polarization is accompanied by distinct changes in metabolic pathways. Agents that influence these processes, including cholesterol metabolism, glycolysis and oxidative stress, are potent therapeutic agents to treat atherosclerosis.
